# Appropriate Dairy Calf Feeding from Birth to Weaning: “It’s an Investment for the Future”

**DOI:** 10.3390/ani10010116

**Published:** 2020-01-10

**Authors:** Laura J. Palczynski, Emma C. L. Bleach, Marnie L. Brennan, Philip A. Robinson

**Affiliations:** 1 Animal Production, Welfare and Veterinary Sciences Department, Harper Adams University, Newport, Shropshire TF10 8NB, UK; ebleach@harper-adams.ac.uk (E.C.L.B.); Philip.Robinson.2@glasgow.ac.uk (P.A.R.); 2 School of Veterinary Medicine and Science, University of Nottingham, Sutton Bonington Campus, Leicestershire LE12 5RD, UK; marnie.brennan@nottingham.ac.uk; 3 School of Veterinary Medicine, College of Medical, Veterinary and Life Sciences, University of Glasgow, Garscube Campus, Bearsden Road, Glasgow G61 1QH, UK

**Keywords:** dairy calf feeding, health, welfare, nutrition, stakeholder attitudes

## Abstract

**Simple Summary:**

Research has indicated that dairy farms often do not feed calves according to recommended best practice, despite legislation and industry advisory efforts. This study used interviews with dairy farmers and their advisors to investigate why farmers feed calves the way they do. Various calf feeding practices were used by participating farmers, largely based on perceived convenience and calf performance. Advisors were concerned that calves were commonly underfed, which may be partly due to farmers receiving inadequate instructions for calf feeding. Our results highlight the need for more consistent and effective recommendations for farmers regarding calf feeding and weaning. Standard guidelines for calf milk replacers should be improved to ensure that calves are fed enough to support basic biological functions and growth. Further research is needed to establish best practices for weaning calves whilst supporting rumen development, health and weight gain. All recommendations for calf feeding should facilitate the achievement of standard industry targets including rearing replacement dairy heifers to calve by 24 months of age.

**Abstract:**

Dairy calves must be fed appropriately to meet their nutritional needs, supporting optimal growth and development to achieve the recommended target age at first calving (AFC) of 24 months. Traditional restricted milk feeding practices suppress growth, contribute to negative welfare states and may result in malnutrition and immunosuppression. Despite more recent recommendations to increase milk allowances for pre-weaned calves, restricted feeding remains a common practice. This study explored the rationales behind the calf feeding protocols used by dairy farmers in England. Forty qualitative interviews (26 farmers, 14 advisors) were conducted between May 2016 and June 2017, transcribed in full, then coded into themes. Results indicate that a variety of calf feeding regimes are used on farms, largely determined by farmers’ attitudes regarding ease of management and the wellbeing of calves. Advisors were concerned about widespread underfeeding of calves, which may be partially due to insufficiently clear recommendations for calf milk replacer (CMR) feeding rates. There was also evidence of uncertainty regarding best practices for weaning calves. Collaboration between academic research and industry is essential to establish a consensus on calf feeding standards which support physiological function, facilitate weaning, support growth targets and ensure calf health and welfare is protected.

## 1. Introduction

Dairy calves must be fed appropriately to meet their nutritional needs for optimal growth and development. Diet must also support and reflect the development of calves’ digestive function from the liquid-fed pre-ruminant phase through the transition into a functional ruminant [1]. There are also financial implications since milk feeding accounts for 40% of total rearing costs from birth to weaning, the most expensive phase of rearing replacement dairy heifers [2,3]. Calf growth rates at least partly determine their age at first calving (AFC), with heifers calving at 23–24 months being more cost-efficient than later calving animals [2]. The recommended target AFC of 24 months achieves optimal economic efficiency resulting from increased lifetime fertility, survival and milk production compared to later calving heifers [4,5,6].

A typical Holstein-type heifer must maintain a growth rate of about 750 g/day from birth to achieve adequate body weight and stature to calve at 24 months [5]. The optimal protein to energy ratio for growth in pre-weaned calves has been estimated to be approximately 11.5 g of crude protein per MJ of metabolisable energy (ME) [7]. Approximately 325 g/day whole milk solids (2.5 L/day) or 380 g/day calf milk replacer (CMR) (3 L/day), which contain about 22.5 MJ ME/kg and 19.5 MJ ME/kg respectively, provide sufficient ME to meet the maintenance requirements of a 45 kg calf under thermoneutral conditions with surplus nutrients supporting growth [1].

Traditional feeding practices provide daily milk allowances of approximately 10% of calf bodyweight, primarily to increase solid-feed intakes to facilitate rumen development for earlier weaning. These restricted feeding practices limit the growth potential of calves [8] and are likely to provide insufficient energy in temperatures below 15 °C [9]. When calves are malnourished, particularly in cases of insufficient energy intakes, their immunity is impaired and they are more susceptible to disease (e.g., [10,11,12]). The effect of feeding higher planes of nutrition, above maintenance requirements, on the immunocompetence of calves is less clear cut as intensive milk feeding does not appear to affect the health and immune status of calves in a consistent manner [12,13].

However, calves will voluntarily consume over 9 L/day of milk [8,14], indicating that larger milk meals are required to satiate calves and improve their welfare. Indeed, restricted milk feeding causes calves to experience persistent hunger, as indicated by higher numbers of unrewarded visits to milk feeders [14,15], more frequent and higher pitched vocalisations [16] and reduced play behaviour [17]. More recent recommendations suggest daily milk or CMR feeds should equate to 20% of calf bodyweight to support calf growth and health [18] and a common target is to have doubled the birth weight of calves by the time of weaning at 8 weeks of age [19]. Increasing the amount of milk or CMR fed per day supports higher growth rates, with the weight advantage persisting post-weaning [20,21], and is linked to developmental effects which positively affect future milk yield [22].

Despite these recommendations, once-a-day milk feeding is sometimes used on farms to reduce labour requirements whilst achieving adequate gains in calf bodyweight [23,24]. In England, The Welfare of Farmed Animals (England) Regulations 2007 and EU Directive 2008/119/EC on the minimum standards for the protection of calves require calves to be fed at least twice-a-day up to six months of age. European legislation also requires that all calves over two weeks of age must be provided with sufficient fresh drinking water to satisfy their needs and have access to water at all times in hot weather or if they are ill. The national legislation in England requires that all calves are provided with sufficient fresh drinking water each day from birth. Once-a-day milk feeding in the first month of life may contribute to abomasal disorders (abomasitis and/or bloat) in calves [25] and is illegal since the limited intakes of solid feed during early life do not constitute a meal. Twice daily milk feeding is necessary to meet calves’ nutritional requirements prior to 28 days of age [25,26].

Water is a key nutrient and plays a critical role in calf growth and rumen development [1] and calves should be provided free access to clean drinking water from birth. Although calves obtain the majority of their water intake through consumption of milk or CMR [27], this water from feed goes directly to the abomasum. Drinking water enters and supports the development of the rumen [28] and encourages greater intakes of starter concentrates [29], milk consumption and growth performance [30].

Despite the research outlined above evidencing the benefits of feeding calves greater milk allowances and offering drinking water from birth, many farms feed a restricted milk diet, and some do not provide access to water prior to weaning [3,31]. Restricted calf feeding has been highlighted as an area of concern in the scientific literature [31,32,33], suggesting that legislation and current industry advisory efforts may have failed to assert best practice on farms. Very few studies have explored the rationale behind the calf feeding systems adopted by farmers. The present study used qualitative interviews to explore the practices, experiences and perspectives of participant dairy farmers and advisors. Such social science approaches are advocated by a growing proportion of the animal health and welfare research community e.g., [34,35,36,37,38]. This paper aims to explore the nuanced reasoning behind the different pre-weaning calf feeding protocols used on English dairy farms to provide greater holistic understanding of the wider context which might influence on-farm decisions.

## 2. Materials and Methods

This study employed a critical realist paradigm which asserts that subjective experiences of phenomena and objective facts are equally important in understanding a topic within its wider context [39]. This epistemology enabled the exploration of different perspectives regarding dairy calf management, providing a more holistic understanding of pre-weaning calf feeding.

### 2.1. Data Collection

Calf management on English dairy farms was investigated through 40 in-depth semi-structured interviews (26 with farmers, 14 with advisors) conducted between May 2016 and June 2017. All interviews were conducted by the first author, a doctoral student who sought to investigate human influences on calf health and welfare regarding rearing practices from birth to first calving. Presented here are findings relating to calf feeding following the provision of colostrum, which has been addressed in a previous paper [40].

Purposive and snowball sampling [41] was used to recruit participants from existing contacts, veterinary practices, dairy events and conferences, and individuals suggested by interviewees. This method yielded farmers who managed a range of dairy herd sizes and production systems (Table 1) and advisors who tended to have a specific interest in dairy youngstock (Table 2). Interviews were grouped according to geographical location with participants from areas of England with high densities of dairy farms (Southwest and Midlands) and from a north-easterly area with less dairy focus in Yorkshire.

Interviewees included 37 dairy farmers (farm managers (n = 17), farm workers (n = 9), calf rearers (n = 8) and herd managers (n = 3)) and 14 advisors (veterinarians (n = 11), feed (n = 2) and a veterinary pharmaceutical company representative (n = 1)). One of three interview formats were used according to participants’ preferences: all advisors and nine farmers were interviewed individually in a seated setting; 20 farmers participated in nine joint interviews where two to three participants were interviewed together; and eight farmers were interviewed whilst walking around the farm.

Two separate interview topic guides were used, one for farmer interviews, the other for advisor interviews. These guides included open-ended questions which ensured interviews remained relevant to calf rearing whilst allowing flexibility to explore areas of most importance to participants [42] rather than being predefined by the researchers. Farmers were asked questions about the practices used on their farm and their opinions about how calves are reared elsewhere, whereas advisors were asked about their main areas of concern regarding calf rearing and their role in providing information and advice. Seven pilot interviews were conducted, four with farmers (F1, F2, F3, F4) and three with advisors (V1, V2, N1) to ensure topic guides were suitable. Responses were useful to the research project and only minor refinements were made to the topic guides so the pilot interviews were included in the overall dataset.

Data collection and analysis overlapped in an iterative approach so that topics raised in earlier interviews could be further examined with later interviewees [43]. Interviews were audio recorded with consent and subsequently manually transcribed in full using f4transkript software (Version 6.2.5 Edu, audiotranskription.de, Marburg, Germany). Data collection ceased when it was judged that thematic saturation was established [43], i.e., the main concepts and range of opinions relevant to calf rearing had been identified, and no new themes were emerging.

### 2.2. Data Analysis

Transcripts were analysed using thematic coding which involved reading and re-reading the data and grouping extracts into common themes [44]. Transcripts were coded in NVivo 11 for Windows (Version 11.4.1.1064 Pro, QSR International Pty Ltd., Victoria, Australia). In first cycle coding excerpts were arranged according to topic, personal values, and processes [43] to inform ongoing interviews and indicate focal subjects including calf feeding. Coding was repeated to explore the topic of calf feeding in-depth and relevant interview extracts were chosen to represent the perceptions of participants relevant to the themes and explanations being constructed.

### 2.3. Ethical Approval

Prior to participation in the study, all participants gave their informed consent—specifically for interviews to be conducted, audio recorded, transcribed, securely stored and for anonymised interview excerpts to be used when reporting findings. The study was conducted in accordance with the Declaration of Helsinki, and the protocol was approved by the Harper Adams University Research Ethics Committee on 13 January 2016 under project number 75-201511.

## 3. Results

Average interview length was 56 min (rage 26–90 min). Most results within the theme of calf feeding pertained to liquid feeds, with some reference to the provision of water and solid feeds in preparation for weaning.

### 3.1. Milk Feeding: Amount Fed

Participating farmers fed their calves 4–8 L milk per day (10 fed whole milk, 16 fed CMR) (Table 3) and the mixing rates, brands and composition of CMR varied. Few farmers could recollect basic details of their CMR, including the protein and fat content. Most farmers provided the weight of CMR fed, since “*water is just the carriage to get [nutrients from CMR] into the calves*” (F8 male, farm manager); the total CMR provided ranged from 500 g–996 g per day, though some farmers referred only to the volume of CMR fed. The majority of milk was fed in two daily feeds unless calves had access to an automatic feeder throughout the day. One organic farm fed cold whole milk ad libitum to calves after the first week. Two farms fed once-a-day milk to calves from 1 to 2 weeks of age and F7 used a particularly concentrated 3 L feed once a day with a mixing rate of 300 g/L, believing that increasing the feeding rate in this manner had improved calf health:

“*Prior to the feeding regime we’re on now I generally tended to restrict milk to 4 L of milk a day, 750 g of milk solids over two feeds, and I would get a lot more enteric disease. I’d get a lot more of all calf health issues*”.

(F7, male farm manager)

Most farmers appreciated that a higher rate of nutrition could contribute to improved calf health and recognised the high feed conversion efficiency for calf growth and potential impacts on future performance. However, several participants believed that on some farms calves were not prioritised as the focus was centred on the milking herd, and advisor participants were concerned that underfeeding of calves was commonplace:

“*The amount of people that feed once a day cold milk to calves despite the fact it’s illegal is still quite high*”.

(V2, female youngstock vet)

“*I think these calves are starved […] The number of people that feed two litres twice a day—which is not even maintenance growth rates, especially considering the [cold] weather*”.

(V3, male youngstock vet)

Farmers seemed less concerned by legislation and calf growth requirements, focusing instead on what suited their management routine and whether calves “*looked well*” (F22, female herd manager). Reasons for restricted feeding included maintenance of traditional practices, following instructions on CMR packaging, and attempts to save money. Calf feeding protocols were usually only changed in response to problems:

“*[On the packaging] 250 g was what was recommended, so that’s what [the calves] got, but they weren’t really doing well on it. You think “it’s disease”, or “it’s the [starter] feed” […] it was actually the lack of a decent amount of milk […] You can’t hide behind saying “I’ll save a bit of money on milk powder” […] it’s an investment for the future*”.

(F5, male farm manager)

That CMR guidelines on commercial product packaging did not provide sufficient nutrition to meet recommended growth targets, e.g., to double the birth weight by weaning, was raised by a veterinarian-turned-feed-consultant (V10):

“*Current recommendations often to a farmer are only about 750 g of milk powder a day […] Even if they’re being as efficient as they possibly could, you’re only gonna get 750 g a day of growth […] and that’s before you factor in any cold or draughty conditions.*”

Furthermore, one farmer (F15) admitted finding instructions to be unclear and fed the same milk solids as a more dilute milk solution when attempting to increase the amount fed to calves (Table 3):

“*Generally it’s just water I’ve been adding […] because reading the instructions on the bag, it doesn’t actually say if you’re supposed to give more powder.*”

### 3.2. Milk Feeding: Type of Milk Fed

The majority of participant farmers (16/26) fed CMR while all participating organic farmers (n = 5) and five conventional farmers fed whole milk (Table 3). Three participants stated that they fed calves unpasteurised non-saleable milk, two fed pasteurised whole milk and five did not specify. Three participants had started feeding whole milk to reduce feed costs during the 2014 downturn in milk prices:

“*I did fall out with my powder milk supplier because the price didn’t come down when milk price came crashing down […] so I put a pasteuriser in. It was expensive […] but the calves are so much better on whole milk than they are on powdered milk*”.

(F13, male farm manager)

Some farmers were very positive about the information and support provided by their feed company representative, and most were willing to invest in “*a feed that’s right*” (F17, male farm hand)—CMR, which was cost-effective rather than the cheapest available. However, what constitutes a ‘good’ CMR was not specified, though some referred to the protein and oil content of their milk powder. Other farmers were distrustful of salespeople and one youngstock veterinarian questioned both farmers’ knowledge of feed components and the ethics of feed companies:

“*If you look at milk powders, some of them, particularly when money was getting very tight, their vitamin E levels suddenly crashed. I think that’s a bit naughty of them [the feed companies] because a lot of farmers won’t really know what’s in their milk powder*”.

(V11, female youngstock vet)

Several participants, particularly organic farmers, perceived feeding whole milk to be more natural and suggested that it resulted in better calf performance, having been “*designed*” (F13, male farm manager) for calf feeding. Feeding whole milk was also considered beneficial in terms of consistency in feeding if more than one person was responsible for feeding calves. Dairy-bred bull and beef-cross calves were either fed the same as dairy replacement heifer calves for ease of management in dual dairy-beef systems or considered to be low-priority “*milk thieves*” (F10, male farm manager) which would be quickly removed from the farm. In these cases, dairy-bred bull calves received poorer-quality feeds, largely due to a poor market value for those calves:

“*I’m rearing a calf, and it’s margin with me […] If they put another £20 worth of milk powder into that calf and get that heifer in-calf three months quicker that’s cheap, but for me it’s £20 directly off*”.

(F8, male farm manager, rears dairy bull calves)

Although feeding waste milk may be standard practice for replacement calves on some farms, unpasteurised non-saleable milk was more commonly fed to bull or beef-cross calves on dairy enterprises.

“*The bull calves and any beef calves, they get […] antibiotic milk, […] high cell count milk, anything really because they’re not going to be around for long enough to pick up anything serious*”.

(F5, male farm manager)

These non-saleable milk feeds often included milk from cows treated with antimicrobials, an area of concern acknowledged by this farm manager:

“*If you’re feeding milk from cows which have been treated with [antibiotics], you’re feeding that antibiotic to those calves. So what problems are you creating? What resistance do you create?*”.

(F19, male farm manager)

### 3.3. Milk Feeding: Preparation and Feeding Method

In addition to what was fed to calves, many farmers emphasised the importance of how milk was prepared and delivered to calves. Farmers using automatic machine feeders believed calves benefited from being able to feed throughout the day:

“*If you’re bottle feeding a calf twice a day, when you feed it it’s always starving and it guzzles it really fast. You don’t get that when they’re on machine because they’re doing it in a more natural way, as if they were on a cow*”.

(F8, female calf rearer)

Automated feeders could also help to ensure consistency of milk feeding, a fundamental principle according to farmer participants. They could also provide farmers with flexible time as they could check the calves when it was convenient rather than being tied to a specific feeding time.

“*If you’re really busy, you don’t have to tend the machines, two or three hours either way, it’s really flexible […] The milk’s always there at the right temperature, it’s well mixed, should be [hygienic] if they’ve kept the machines clean*”.

(F21, male farm manager)

However, the cost of machine-feeders prevented many farms from installing them.

Several participants stressed the need for staff to have the time and equipment available to make calf feeding easy and simple to facilitate proper feeding. However, mixing CMR involves several variables, including water temperature, mixing rates and timings, and if the person responsible for calf feeding does not use measuring implements or if several people feed the calves, consistency may suffer and affect calf performance.

“*I use a thermometer and I mix at 40 °C and I feed at about 38 °C. Dad uses his finger and I couldn’t tell you what [temperature] he feeds at […] Then concentration, I’ve given him a scoop that’s pretty failsafe, but when I was doing it myself I did get better results*”.

(F19, male farm manager)

Teat feeding was considered beneficial by most farmers. Some had made the change from bucket feeding and were impressed with the results, or acted on external information:

“*One journal said that teat feeding over bucket feeding actually helps them grow a little quicker […] I’m not sure if it does, but I tried doing it anyway*”.

(F3, male farm hand and calf rearer)

“*[I visited a farm with stunning calves, the farmer] said whatever you do, do not feed a calf on a bucket. It gulps it down, it gets into the wrong stomach. He said, when a calf suckles, it produces saliva, you can see it around its mouth, that aids digestion*”.

(F8, male farm manager)

However, one farm veterinarian indicated that the feeding position resulting from the angle of teats on bar feeders may contribute to respiratory disease:

“*I think calves on a bar feeder get a certain degree of aspiration pneumonia from the teats being horizontal […] I can’t understand why no one’s invented a calf bucket that’s got like a corner cut off and the teat coming out on the 45° angle so that it forces them into a neck down, head up position which is more natural*”.

(V4, male farm vet)

Hygiene of the feeding equipment was considered important by both farmer and advisor participants to foster good calf health.

“*[Calves] are babies. You have to keep your bottles clean, disinfect everything in-between feeding each calf on a bottle […] even if they’re healthy calves, I always disinfect the teat*”.

(F18, female calf rearer)

However, cleaning may not be done to a high standard on farms and may not be recognised as a problem by farmers:

“*[I recommended increasing] everyone’s milk that they were feeding, and everyone would say “oh no, if I do that they scour!” […] I think it was just general hygiene of the milk preparation and the buckets. So when they cleaned that, adding more milk wasn’t the problem*”.

(V11, female youngstock vet)

Advisors tended to attribute lack of hygiene to farm facilities and poor availability of hot water. Reasons given by farmers for a lack of hygiene in calf feeding included lack of perceived efficacy in disease control and a perception that sanitation hinders the acquisition of immunity:

“*Some people say you should disinfect between [feeding groups of calves], but I never have done. If one lot gets [an infection], they usually all get it anyway*”.

(F14, male calf rearer)

“*Everything should be washed and sterilised with hot water after every calf’s fed. With that you’re not giving the calf the chance to build up any immunity*”.

(F16, male farm manager)

### 3.4. Solid Feed, Weaning and Water

A range of weaning methods were implemented by farmers, although the majority were weaning calves at around 7–8 weeks (Table 4). Some based weaning decisions on age alone whilst others considered calf weight or starter intakes. There was generally a negative view of early weaning practices:

“*It seems to me there’s this race to wean the calves as quickly as you can. “We wean all calves at six weeks old.” It’s unnatural. […] You’re gonna grow better animals by just feeding them milk for longer*”.

(F16, male farm manager)

Farmers fed calves different starter feeds and forage, and used different methods for gradual weaning. Some decreased the volume or concentration of milk fed, others decreased the number of daily milk feeds. One farm veterinarian (V4) admitted being unsure of the ‘best’ weaning technique:

“*Weaning, I don’t think there’s a right answer with that. I certainly haven’t found it yet […] How you reduce the milk? Some people will do it by going from two times a day to once a day. Some people will continue twice a day, feeding smaller amounts. Some people will continue twice a day, feeding the same amount but a lower concentration and I don’t know what the right answer is to be honest with you.*”

Participants were aware that calves should be consuming solid feed and forage to aid rumen development, and milk feeding practices sometimes needed to be altered to facilitate intakes of dry starter.

“*We do struggle to get roughage in them […] We’ve had the odd post-mortem done on calves which have been poor and we’ve had poor rumen development so it’s something we’re trying to improve on*”.

(F9, male farm manager)

“*We tried a kilogram [of CMR] a day, but we found that although the calves looked great at weaning time, they didn’t wean as well. I don’t think they had room to eat as many pellets. This way [875 g/day], they eat more pellets and it’s a more seamless weaning*”.

(F10, male farm manager)

Problems encountered at weaning time included pot-bellied calves, growth checks and diarrhoea. Some farmers had changed their practices and improved weaning, whereas others struggled to prevent problems, despite trying several alterations in a trial-and-error approach:

“*I used to wean everything at six weeks. We’d go once a day milk at five weeks and they’d be weaned at six. But now we do twice a day feeding until six weeks and then once a day for another two weeks, monitoring how much corn they’re eating. By eight weeks old they’re taking a lot of corn, and then we wean them. That’s made quite a difference to the calves in that they used to be pot bellied and horrible after weaning, but they’re not now*”.

(F5, male farm manager)

“*[The calves] do get very loose [at weaning] and that’s mostly when the coccidiosis kicks in […] I know you shouldn’t do everything all at once. They’re trying to be weaned, they’re changing the ration, they’re introduced onto silage—that’s when they get loose. I’ve tried not giving them silage, I’ve tried keeping them on pellets, I’ve tried putting them on rearing nuts [sooner] and they still get loose, so it doesn’t really seem to make a lot of difference*”.

(F14 male calf rearer)

Water affects calf consumption of concentrates, plays an important role in rumen development and its provision is required under UK and EU law. However, many advisors were frustrated that calves on many farms did not have access to fresh water.

“*You can walk around quite a lot of dairy farms in the UK that the calves don’t have access to water. The fact that it’s illegal let alone detrimental to growth rates…*”.

(V2, female youngstock vet)

“*[Farmers will] complain to you “oh, they’re not eating much dry starter feed, your feed’s rubbish”—you’re not really gonna want to eat dry crackers without a drink of water, are you? They don’t realise that [calves] need fresh water for rumen development. Their milk feeds twice a day—it doesn’t constitute free water. It doesn’t go to the rumen for rumen development—it goes to the abomasum*”.

(N2, female feed company calf specialist)

Some farmers who did not provide water to young calves believed that calves would reject their milk feed after gorging on water, particularly if both were provided in buckets rather than milk via a teat. Others did not realise that calves required access to free water in addition to their liquid feeds.

“*One thing is that they don’t fill up on water, so when you feed them they’re hungry enough to drink the milk. They shouldn’t really need it. It’s like a newborn baby, you don’t give them water. Apart from warm milk, they don’t need anything else*”.

(F16, female calf rearer)

“*Milk when you feed it is a fixed dry matter content and fixed fat and protein content, so you haven’t got the element of a thirst-quenching feed for the baby calf*”.

(GA1, female government veterinary advisor)

If calves seem to be doing well, often practices are not altered and farm staff may not have control over management decisions.

“*This is a source of contest between me and the bosses because I think they should have water all the time, but they only feed water when they get to about a month old […] that’s how they’ve always done it, and the calves look really well so I can’t really tell them to do otherwise*”.

(F22, female herd manager)

## 4. Discussion

Our results indicate that a wide variety of calf feeding regimes, primarily to rear replacement heifers, are used on English dairy farms. Whilst participant farmers reported providing concentrates and forage to calves, discussion in our interviews was focused on liquid feeding, particularly CMR. Farmers’ actions concerning calf feeding practices were largely determined by their attitudes regarding the ease of management and wellbeing of calves. Some farmers made proactive changes seeking to achieve optimal calf performance, with several noting the benefits of feeding programmes which promote accelerated growth. Most participants maintained the status quo, continuing historic practices, including limiting liquid feed allowances and only making alterations in response to perceived problems with calf health or growth rates. However, farmers may struggle to accurately assess calf performance due to a lack of calf monitoring data [45], possibly resulting in failure to identify problems. Calf feeding is also often regarded as a simple, childhood task that does not require discussion or deliberation, particularly if calves are perceived to be performing well [46].

In the present study, advisors, particularly veterinarians, were concerned about widespread underfeeding of dairy calves. Sumner and von Keyserlingk [33] found that Canadian dairy cattle veterinarians were also concerned about calf hunger and malnutrition, suggesting that underfeeding calves is potentially a global problem in the dairy industry in developed countries. This may, at least in part, be due to the long-established industry standard for restricted milk feeding which has only relatively recently been challenged to favour greater milk allowances for improved calf performance [18,20,21,22] and better welfare standards [8,14]. However, it has also recently been argued that increasing intakes of solid feed during the pre-weaning period alongside appropriate liquid feeding (as opposed to accelerated liquid feeding programmes) offers a more cost-effective route to achieving greater growth rates whilst also supporting rumen development and future lactation performance [47]. This lack of consensus in the research literature is reflected by the range of milk allowances provided by participant farmers. Farmers were providing approximately 5–6 L/day of liquid feed to calves on average, with most feeding above the historically-favoured daily rate of 4 L/day. However, the traditional practice of restricted milk feeding persists on many farms [3,31], including a minority of those participating in this study. Several farmers had increased the milk allowance for calves and perceived the change positively, largely pertaining to improved calf health. This indicates that their previous milk ration did not provide calves with sufficient nutrition, impairing their immune function [12,13], and increasing liquid feed allowances covered this nutritional deficit.

Contrary to the legislative requirements, once-a-day milk feeding for young calves was used on two farms in this study. One farm was a rearing unit for dairy bull calves seeking the most time- and cost-effective feeding method for their calves. The other farmer provided the recommended daily milk solids to replacement heifer calves in one highly concentrated feed (30% CMR solution) and observed improved calf health as a result. However, these perceived health benefits are again likely due to the provision of increased nutrition compared to the previous restricted feeding programme rather than the provision of a single, concentrated daily feed. Calves can digest large milk meals of up to 6.8 L (13.2% of bodyweight) without evidence of abdominal discomfort or milk entering the rumen [48]. However, large, infrequent milk meals can cause negative metabolic changes including impaired insulin sensitivity which may negatively affect animals long-term [49]. Despite the legal requirement to provide two liquid meals per day to calves under 28 days of age, some CMR products have been marketed as being suitable for once-a-day feeding [25], thereby encouraging it as an acceptable protocol on farms.

The ethics or technical competency of some animal feed companies was questioned by some of the participants in this study. In particular, concerns were raised that recommended feeding rates from manufacturers of CMR may not facilitate optimal growth efficiency. Calves fed high rates of CMR can achieve growth rates of 1 kg/day [8], but a recent study showed that normal pre-weaning feeding practices on commercial farms resulted in 70% of calves failing to achieve the recommended growth rate of 0.7 kg/day, and 20% of those calves grew at less than 0.5 kg/day [50]. That study did not report how the participating farms established their feeding protocols, but it is likely that current industry standards which may not be based on the optimal physiological requirements of calves [50] contribute to the consistent failure to meet the recommended AFC of 24 months [51]. It is imperative that recommended feeding rates are sufficient to meet calf nutritional requirements and support growth rates which are compatible with industry targets, and that product packaging is updated to reflect these recommendations.

The current study also raises concerns about the clarity of the instructions provided on CMR product packaging, as written instructions for mixing CMR with water to obtain the correct concentration for calf feeding were misunderstood by at least one farmer in the present study. Farmers respect the advice given by trusted feed company representatives who are familiar with their farm and the farms of others [52] so in-person advice which can account for farm-specific rearing targets may be the best way to facilitate optimal feeding protocols on farms. Regardless, written instructions for preparing liquid feeds to pre-weaned calves should be easy to follow in order to support farmers who do not accept in-person advice, and to act as a reference or reminder when mixing CMR at calf feeding.

Few participant farmers accurately measured the temperature of the liquid mix or the amount of CMR included in the feed provided to calves. A consistent liquid diet is important for calf performance; inconsistent provision of milk solids hinders growth, starter intake and feed efficiency [53]. Whilst most farmers appreciated the need for consistency in calf feeding systems, it could be difficult to achieve in practice, largely affected by the values and priorities held by the person responsible for calf feeding, but also the time, equipment and facilities available. Despite the importance of stockmanship [54], most studies have focused on the feeding systems employed by farms, rather than the individuals employing them (e.g., [3,55]). This study indicated that designated calf rearers tended to be most diligent regarding calf feeding, prioritising attention to detail including measuring the variables affecting CMR feeding consistency. On farms where calf feeding was carried out by persons with other responsibilities on the farm, feeding processes were more variable, possibly stemming from a lack of time dedicated to calves and a sense of diminished responsibility compared to designated calf rearers. Automated milk feeders were useful calf management aids for the farms that had them, and can improve welfare due to calf socialisation and constant access to feed which is consistently mixed and at an appropriate pre-set temperature. However, machine feeders have high upfront capital costs, require suitable accommodation for grouping calves, and may contribute to increased disease incidence due to the hygiene challenges presented by calves sharing a single teat [56].

Good hygiene regarding food preparation was prioritised to varying degrees on farms; some diligently disinfected equipment between feeding each calf or pen, others did not. This was sometimes due to pessimistic perceptions that hygiene was ineffectual in disease control, but management problems including uncleanliness have been shown to contribute to increased rates of diarrhoea [57,58]. Others believed sterilisation hindered the acquisition of immunity, similar to misunderstandings previously reported in areas of colostrum management [40] and biosecurity [37]. Indifference or negative attitudes towards ensuring good hygiene are problematic since sanitary feeding equipment and accommodation are critical to maintaining good calf health [18,56]. Furthermore, such attitudes may compound the restricted feeding of calves, as indicated in the literature [18] and by a youngstock veterinarian in the present study, who revealed that farmers often associated increased milk allowances with increased incidences of diarrhoea in calves, but cases of calf scour were more likely to stem from poor hygiene.

In addition to the contribution of poorly sanitised feeding equipment to calf ill-health, one veterinarian in the current study believed the angle of artificial teats on bar feeders could cause aspiration pneumonia in calves. The authors are not aware of research investigating this issue, since aspiration pneumonia is more commonly associated with incorrect oesophageal feeding [59,60] but if proven, calf feeders may need to be adapted and their design improved to encourage correct feeding position and reduce the risk of aspiration. Artificial teat feeding is recommended to allow expression of natural sucking behaviour and aid digestion [58] through activation of the oesophageal groove reflex which bypasses the rumen for milk to enter the abomasum. Farmer participants appreciated this, referencing milk entering ‘the wrong stomach’ in the absence of a teat and saliva.

Feeding unpasteurised whole milk, or non-saleable milk, can also contribute to pathogenic risk [1]. Of the nine participating farmers feeding whole milk to calves, only two stated that they pasteurised whole milk before feeding it to calves, one of whom was using waste milk, and a further two participants fed unpasteurised non-saleable milk. The practice of feeding milk from cows treated with antimicrobials is also a key area of concern in relation to antibiotic resistance [61] as antibiotic residues cannot be decreased through pasteurisation. Also, feeding milk containing antimicrobial residues causes microbial imbalance in the gut microbiome of pre-weaned calves [62]. These issues appear to be most common in relation to bull or beef-cross calves from dairy enterprises due to the cost of feeding CMR or saleable milk, but some farms also fed their dairy heifers non-saleable milk as standard practice. This could be because the up-front cost of installing a pasteuriser is considered prohibitive or the benefits of pasteurisation and the risks of feeding non-saleable milk are not well understood by farmers, suggesting a need for proactive advice from veterinarians.

The information interviewees provided regarding their CMR lacked detail. Whilst farmers would refer to the need to use a ‘good’ feed, they did not provide definition. This suggests that farmers require further guidance on calf nutrition, and it is likely that they relied heavily upon the information provided by their feed merchant or product packaging. The current study relied only on interviewee accounts which limited our ability to precisely assess what was fed to calves. However, detailed analyses of feed packaging or written records were beyond the scope of the study. The interviews did provide a useful overview of calf feeding and highlighted a potential disconnect between current recommendations and information provided on CMR packaging as outlined above. The interviews also showed that participants were most focused on liquid feeding of calves, with limited discussion of concentrate and forage feeding for milk-fed calves beyond ensuring adequate intakes of dry feed prior to weaning. Young calves are most at risk of diarrhoea and mortality [63], and there are arguably more variables and effort involved in providing milk or CMR to calves (temperature, consistency, timing, feeding method, hygiene) compared to providing calf starter and roughage. Participants said very little about the post-weaning feeding of calves, attitudes which are reflected in the lack of coverage of the post-weaned period to approximately 4–5 months of age in the research literature [64].

Participants’ main focus regarding dry feed for calves was ensuring adequate intakes to prepare calves for weaning. All producers in this study used some form of gradual weaning, and none weaned earlier than six weeks of age. Farmers mainly based weaning decisions on calf age, with some also considering calf bodyweight or starter intake, recognising that calves should be consuming over 1 kg/day of dry calf starter before weaning to indicate sufficient rumen development and prevent growth checks [1]. These practices should support gastrointestinal growth and development in dairy calves [65]. However, not all farmers provided calves with access to water from birth, which may negatively affect rumen development, restricting pre-weaning feed-efficiency and impeding growth both pre- and post-weaning [30]. This could be related to the poorly described water requirement for calves and few published research articles which include calf water intakes [64].

Furthermore, the range of weaning practices used on farms indicates that there is a lack of consistent guidance regarding the best way to wean calves, or if there is, it is not being consistently implemented at farm level. Research has largely focused on the positive effects of gradual weaning based on concentrate intakes [66] and the effect of pre-weaning milk or CMR allowances on the weaning and post-weaning period [67]. However, participants were unsure of the best weaning methods, largely pondering whether transition should be done by diluting milk feeds, reducing the number of feeds, or reducing the quantity fed at each meal. Even a veterinarian who would be expected to have a good understanding of the developing bovine digestive physiology was unsure which weaning method was most effective. This suggests the industry requires further evidence-based recommendations for practical methods to wean calves, particularly *how* to reduce milk provision to best transition calves onto solely solid feed. Several participant farmers also reported that calf health status and growth rates were most problematic at weaning time, suggesting their calves did not have sufficiently-developed rumens when transitioned from milk to solid feed, or that forage intakes are insufficient to mitigate ruminal acidosis [68] and support the establishment of diverse rumen bacteria [69]. Our results indicate a need for further research to establish a consensus on optimal weaning techniques so that farmers can be more effectively advised.

In summary, there is considerable variation in the calf feeding practices used on UK dairy farms, possibly reflecting the current lack of consensus in the scientific literature regarding the most cost-effective feeding protocols to promote growth and future performance. Although now outdated, restricted milk feeding was the predominant recommendation for decades, and advice must be consistent and have evident benefits at the farm level to shift mindsets away from restricted milk feeding. Some CMR feed manufacturers may need to review their feeding recommendations in order to better ensure calves’ nutritional needs are met. More consistent advice, for example, about the importance of drinking water and hygiene practices regarding milk feeding, have also not stimulated all farmers to implement best practice. In these cases, it is possible that more effective calf performance monitoring and peer-to-peer learning may help to show farmers that their methods may not be as efficient as they could be, thus motivating them to make improvements [46].

Farmers would also likely benefit from more input from their advisors to counter the variation and confusion about what to feed calves and how to do it. However, it appears that the area of calf nutrition is somewhat of a grey area in terms of advice. Veterinarians may not be focused on the calf rearing of their dairy farm clients [33] and are often not asked by the farmers about calf feeding. It might seem more appropriate to seek advice from trusted animal nutritionists or feed merchants [70], though some participants in this study indicated they would be distrustful of receiving a sales pitch rather than honest information about the best way to feed their calves. Collaboration between veterinarians and the feed industry could help to improve the consistency of recommendations for ensuring suitable calf nutrition. Working together, veterinarians, feed merchants and nutritionists could offer farmers high-quality, bespoke advice about the most cost-effective nutrition and feeding systems that would provide for the health and wellbeing of calves on individual farms.

## 5. Conclusions

Feeding practices on dairy farms tended to be based on perceived calf performance, and the simplicity, efficiency and cost- or time-effectiveness of their feeding practices versus potential alternatives. However, farmers cannot be expected to implement best practice if the recommendations for standard feeding provide insufficient nutrition and guidance regarding weaning protocols. The advice available to farmers on the subject of practical calf feeding needs to be improved and communicated by advisors. In particular, the animal feed industry should make a more concerted effort to ensure guidelines are compatible with the physiological needs of calves, facilitate weaning and support growth targets to achieve earlier AFC.

## Figures and Tables

**Table 1:** Farmer participant demographics.

Interview Code, Style	Interviewee Details: Job, Gender, Age Estimate	Farm Details: Calving Pattern, Herd Size, Farm System	Location within UK
F1, Mobile	Calf rearer, f, 20–30	AYR, 380, conventional	Midlands
F2, Sit-down	Calf rearer, f, 40–50	AB, 350, conventional	Midlands
F3, Sit-down	Farm hand/calf rearer, m, 20–30	AYR, 350, conventional	Midlands
F4, Joint	Farm manager, m, >50 Farm hand, f, 20–30 Son/trainee vet, m, 20–30	AYR, 120, conventional	Midlands
F5, Sit-down	Farm manager, m, >50	AB/SB, 70, conventional	Midlands
F6, Sit-down	Calf rearer, f, 30–40	SB, 300, organic	Midlands
F7, Mobile	Farm manager/calf rearer, m, 30–40	AYR, 280, conventional	Midlands
F8, Joint	Farm manager, m, 40–50 Farm wife, f, 40–50	Dairy bull calf rearer, batches of 20 calves	Yorkshire
F9, Mobile	Farm manager, m, 40–50	AYR, 250, conventional	Yorkshire
F10, Mobile	Farm manager, m, >50	AB, 90, conventional	Yorkshire
F11, Mobile	Farm administrator, f, 30–40	AYR, 400, conventional	Yorkshire
F12, Joint	Farm manager, m, 40–50 Herd manager, m, 20–30	AB, 370, conventional	Yorkshire
F13, Sit-down	Farm manager, m, >50	SB, 600, conventional	Southwest
F14, Joint	Farm manager, m, >50 Calf rearer, m, 40–50	AB, 420, organic	Southwest
F15, Joint	Farm manager, m, 30–40 Calf rearer, m, 30–40	AYR, 120, conventional	Southwest
F16, Joint	Calf rearer, f, 30–40 Farm manager, m, 30–40	SB, 250, organic	Southwest
F17, Joint	Farm manager, m, >50 Farm hand, m, 20–30 Farm hand, f, 20–30	Dairybull/beef calf rearer, 1400 calf places	Southwest
F18, Sit-down	Calf rearer, f, 20–30	AYR, 180, conventional	Southwest
F19, Sit-down	Farm manager, m, 30–40	AYR, 160, conventional	Southwest
F20, Sit-down	Farm manager, m, 30–40	AB, 330, conventional	Southwest
F21, Mobile	Farm manager, m, 40–50	AYR, 1200, conventional	Yorkshire
F22, Mobile	Herd manager, f, 20–30	AYR, 130, conventional	Yorkshire
F23, Mobile	Farm hand/calf rearer, m, 30–40	AB, 250, organic	Southwest
F24, Sit-down	Herd manager, m, 20–30	AYR, 200, conventional	Southwest
F25, Joint	Farm manager, m, >50 Calf rearer, m, 20–30	AYR, 350, organic	Southwest
F26, Joint	Farm manager, m, >50 Calf rearer, f, >50	AB, 500, conventional	Southwest

Abbreviations: male (m), female (f), all-year-round calving pattern (AYR), autumn block calving pattern (AB), and spring block calving pattern (SB).

**Table 2:** Advisor participant demographics.

Interview Code, Style	Interviwee Details: Job, Gender, Age Estimate	Location within UK
N1, Sit-down	Feed company salesperson, m, 40–50	Midlands
N2, Sit-down	Feed company calf specialist, f, 30–40	Midlands
DR1, Sit-down	Pharmaceutical company veterinary advisor, f, 30–40	Midlands
GA1, Sit-down	Government veterinary advisor, f, 40–50	Southwest
V1, Sit-down	Veterinary specialist in cattle health, m, 30–40	Midlands
V2, Sit-down	Youngstock veterinarian, f, 20–30	Midlands
V3, Sit-down	Veterinarian starting a youngstock discussion group, m, 20–30	Yorkshire
V4, Sit-down	Farm veterinarian, works on beef calf rearing unit, m, 20–30	Yorkshire
V5, Sit-down	Practice director and youngstock veterinarian, m, 30–40	Southwest
V6, Sit-down	Youngstock veterinarian, m, 30–40	Southwest
V7, Sit-down	Practice partner and farm veterinarian, f, 40–50	Southwest
V8, Sit-down	Practice partner and farm veterinarian, m, >50	Southwest
V10, Sit-down	Out of practice veterinarian, now feed consultant, m, 40–50	Midlands
V11, Sit-down	Youngstock veterinarian, f, 30–40	Southwest

Abbreviations: male (m), female (f).

**Table 3:** Information given by farmer participants regarding heifer calf milk feeding.

Farm	Colostrum	Milk Feeding			
		Type	Amount Per Day	Feeding Method	Temperature
F1	1 feed of 4 L	CMR	2.8 L twice daily	Teat bottles filled from mixer	40 °C set on equipment
F2	2–3 days: 4 L first feed then 2.5 L twice daily	CMR (26% CP)	3.5 L twice daily (2.5 L twice daily first week)	Multi-teat bucket feeder filled from mixer	40 °C set on equipment
F3	4 days: 2 L twice daily	CMR	3 L twice daily (166 g/L) (2 L over 2 weeks)	Multi-teat bucket feeder	Warm (not measured)
F4	3–4 days: 3 L first feed, then amount not stated	Waste WM	Not stated	Multi-teat bucket feeder	Warm (not measured)
F5	4 days: amount not stated	CMR (26% CP)	400 g milk solids twice daily	Multi-teat bucket feeder	Warm (not measured)
F6 ^2^	3–4 days: 3–5 L first feed, left with dam for 24 h then 3–4 L twice daily	WM (Johne’s-free only)	3–4 L twice daily	Via teat	Warm, straight from parlour
F7	“As much colostrum as I can get it to drink”	CMR (26% CP, 20% oil, skim-based)	3 L once daily (300 g/L) (3 L twice daily 150 g/L until day 7–14)	Teat bottles filled from mixer	Not stated
F8 ^1^	Calves not on farm at this point	CMR (whey-based)	Total amount not stated, 150 g/L	Automated feeders with teat	Warm, set on feeder
F9	2–3 feeds of 3 L	WM, soon CMR again ^3^	Not stated	Multi-teat bucket feeder	Warm, straight from parlour
F10	2 feeds of 3–4 L	CMR (skim-based)	3.5 L twice daily (125 g/L)	Not stated	Warm (measured on thermometer)
F11	1 feed of 3 L	CMR (skim-based)	6 L over the day (150 g/L)	Automated feeders with teat	Warm, set on feeder
F12	2 feeds, amount not stated	WM ^3^	Not stated	Multi-teat bucket feeder	Warm, straight from parlour
F13	1 feed of 2 L	Pasteurised waste WM ^3^	Not stated	Multi-teat trailer feeder	40 °C from pasteuriser
F14 ^2^	One feed then left with dam for 24–48 h	Pasteurised WM	3 L twice daily	Multi-teat bucket feeder	Warm from pasteuriser
F15	One feed of 2–4 L then left with dam for 3–4 days.	CMR	2.5 L twice daily (100 g/L) (2 L twice daily, 125 g/L until day 9)	Multi-teat bucket feeder	38–40 °C
F16 ^2^	Left with dam for 24 h	WM	*Ad libitum* (3 L twice daily first week)	Multi-teat buckets, barrels or trailer feeder according to group size	Warm for first week, then cold
F17 ^1^	Calves not on farm at this point	WM	3L once daily (125 g/L) from arrival date (14 days of age)	Trough (no teats) filled from mixer	Not stated
F18	6 L within six hours of birth	CMR	Not stated	Teat bottle for first couple of weeks then bucket (no teat)	Not stated
F19	Left with dam for 24–48 h. Two 3 L feeds if necessary	CMR	3 L twice daily (150 g/L)	Not stated	38–40 °C measured using thermometer by interviewee, but not others
F20	2 feeds of 2.5–3L	CMR (50% skim)	Not stated, but decrease to once daily feeds at 3 weeks	Multi-teat bucket feeder	35 °C
F21	1 feed of 4 L	CMR	6 L over the day (150 g/L (increased from 4.5 L first couple of weeks)	Bucket fed for 10 days then automated feeders with teat	Warm, set on equipment
F22	Left with dam for 3 days. Will feed if necessary	WM	2 L twice daily	Bottle fed for first few days then bucket fed	Warm (not measured)
F23 ^2^	Left with dam for a week	Waste WM	3 L twice daily	Multi-teat bucket feeder	Warm from parlour
F24	1 feed of 2.5–3 L within six hours	CMR	3 L tiwce daily (166 g/L) (increased fro 2 L first week)	Multi-teat bucket feeder	Not stated
F25 ^2^	2 feeds of 2–3 L within 24 h	Waste WM	2 L until 3–4 weeks, then 2.5 L twice daily	Multi-teat bucket feeder	Warm
F26	2–3 feds within 24 h, amount not stated	CMR	Up to 7 L over the day (137 g/L)	Automated feeders with teat	Warm, set on feeder

Abbreviations: calf milk replacer (CMR), whole milk (WM), crude protein (CP). ^1^ Rears dairy bull or beef cross calves. ^2^ Organic. ^3^ Price driven decision. Any details not included in the table mean those aspects were not covered during the interview.

**Table 4:** Information given by farmer participants regarding weaning practices.

Farm	Water	Solid Feed	Weaning Process	Calf Weight Recording
F1	From birth	Rearing pellets from birth	Gradual when calve weighs 80 kg and consume 1 kg starter	Weekly from birth using weigh-crate. Aim for 0.8–0.9 kg/d growth
F2	From birth	Corn and straw from birth	Decrease to one daily milk feed at 7–8 weeks. Weaned when consuming 2 kg starter	At turn-out (6–7 months). Plan to improve weigh system
F3	From birth	Rearing pellets from birth	Group housed at 6 weeks to begin weaning by decreasing volume or concentration of milk	No. lacks time. Mental record of intakes and growth
F4	Not stated	Straw and concentrates from a week old	Gradual decrease in milk concentration between 6–10 weeks depending on availability of milk and intakes of concentrates	No. Judge by end product (target AFC 24 months)
F5	Not stated	Corn	Decrease to one daily milk feed at 6 weeks, weaned at 8 weeks depending on availability of milk and intakes of concentrates	No. Judge by end product (target AFC 24 months)
F6 ^2^	Not stated	Rearing nuts, oats, straw from birth	Gradual decrease in volume of milk at each feed. Weaned at 12 weeks (organic standard)	At movements between accommodation and vaccinations. Aim for 0.8 kg/d growth.
F7	From birth	Rearing pellets (18% CP) from birth	Decrease volume of milk according to intakes of dry starter feed not based on age. Weaned when consuming 2 kg starter for one week	Used to. Established regime that achieved desired growth rates. Aim for >850 g liveweight gain by calving (target AFC 24 months)
F8 ^1^	Not stated	Rearing pellets, home mix (barley, distillers grains, soya, rape meal and minerals), straw	Automated feeders programmed to decrease volume of milk allowance	No. Intends to start
F9	From birth	Rearing pellets from birth and straw from three weeks	Weaned at 8–10 weeks, later if calf is small	No. Labour intensive. Plan to incorporate automated weigh system
F10	From birth	Rearing pellets and straw from birth	Weaned over the course of a week at 7–8 weeks when calf weighs 80–85 kg	Girth measurements at birth and before weaning at 7 weeks. Aim to double birth weight by weaning
F11	Not stated	Concentrates, home mix	Automated feeders programmed to reduce milk allowance by 0.2 L/d day 40–65	Girth measurement at birth Weigh scale output manually recorded periodically. Aim to double birth weight by weaning.
F12	Not stated	Minimal concentrates, grass	Weaned at about 12 weeks when calf weighs 100 kg	Weighed when approaching weaning and about a month after weaning. Compare annual average values.
F13	Not stated	Minimal concentrates, barley, grass	“we probably keep them on milk a little bit longer than we need to”	No. New employee to take groups of calves over local weighbridge
F14 ^2^	First week	Rearing pellets	Decrease milk from 7–12 weeks	Monthly weights taken to calculate growth rate
F15	First week	Rearing pellets, barley straw or hay	Decrease to one daily milk feed at 6–7 weeks for one week	Not stated (Targed AFC > 24 months)
F16 ^2^	Four weeks	Straw, grass, no concentrates	Decrease to one daily milk feed of decreasing volume to wean at 12 weeks	Not stated
F17 ^1^	From arrival	Concentrates, straw	Start weaning when calf weighs about 80 kg	Weighed on arrival and departure over local weighbridge
F18	From birth	Rearing nuts, barley straw	Decrease to one daily milk feed at 6–7 weeks for one week before weaning at 7–8 weeks, depending how calf is doing	No. Intends to start
F19	From birth	Concentrates and straw first week	Weaned at 12 weeks	Girth measurements taken throughout rearing period
F20	From birth	Rearing pellets, chopped wheat straw	Weaned at 8–9 weeks	No. Wants a simple, easy system to use
F21	Not stated	Rearing pellets, straw	Automated feeders programmed to reduce milk allowance by 0.6 L/d day 49–59	Periodically. Would like vet-tech service to reduce labour cost
F22	Four weeks	Rearing nuts, hay	Not stated	No. Does not seem feasible or small farms
F23 ^2^	Three weeks	Rearing pellets, straw	Weaned at 12 weeks	No. Would like to start but can judge by eye
F24	Not stated	Concentrates	Weaned at 8–10 weeks	No. Intends to start
F25 ^2^	Not stated	Rearing pellets	Decrease to one daily milk feed from 10–12 weeks	Regular use of weigh-crate
F26	From birth	Concentrates, straw	Weaned at 7–9 weeks. Automated feeders programmed to decrease volume of milk allowance.	Not stated

Abbreviations: age at first calving (AFC), crude protein (CP). ^1^ Rears dairy bull or beef cross calves. ^2^ Organic. Since no quantitative survey of farm practices was conducted, some details were not included in the interviews—this does not necessarily indicate that calves were not provided with components e.g., straw, water. Straw is stated where it is provided as a feed substrate rather than as bedding.
